# *Gloeothece* sp.—Exploiting a New Source of Antioxidant, Anti-Inflammatory, and Antitumor Agents

**DOI:** 10.3390/md19110623

**Published:** 2021-11-04

**Authors:** Helena M. Amaro, Rita Barros, Tânia Tavares, Raquel Almeida, Isabel Sousa Pinto, Francisco Xavier Malcata, Ana Catarina Guedes

**Affiliations:** 1 CIIMAR—Interdisciplinary Centre of Marine and Environmental Research, University of Porto, Terminal de Cruzeiros de Leixões, Av. General Norton de Matos, s/n, 4450-208 Matosinhos, Portugal; lena.amaro@gmail.com (H.M.A.); isabel.sousa.pinto@gmail.com (I.S.P.); 2 i3S—Institute for Innovation and Health Research, University of Porto, Rua Alfredo Allen, 208, 4200-135 Porto, Portugal; rita.barros@gmail.com (R.B.); ralmeida@ipatimup.pt (R.A.); 3 IPATIMUP—Institute of Pathology and Molecular Immunology, University of Porto, Rua Júlio Amaral de Carvalho, 45, 4200-135 Porto, Portugal; 4 FMUP—Faculty of Medicine, University of Porto, Alameda Prof. Hernâni Monteiro, 4200-319 Porto, Portugal; 5 LAQV-REQUIMTE, Department of Chemical Sciences, Faculty of Pharmacy, University of Porto, Rua Jorge Viterbo Ferreira, 228, 4050-313 Porto, Portugal; tsgtavares@gmail.com; 6 LEPABE—Laboratory of Engineering of Environmental, Biotechnology and Energy Process, Rua Dr. Roberto Frias, s/n, 4200-465 Porto, Portugal; fmalcata@fe.up.pt; 7 FCUP—Faculty of Science, University of Porto, Rua do Campo Alegre, s/n, 4169-007 Porto, Portugal; 8 Department of Chemical Engineering, University of Porto, Rua Dr. Roberto Frias, s/n, 4200-465 Porto, Portugal

**Keywords:** lutein, β-carotene, linolenic acid, linoleic acid, lipidic compounds, carotenoids, PUFAs

## Abstract

Bioactive lipidic compounds of microalgae, such as polyunsaturated fatty acids (PUFA) and carotenoids, can avoid or treat oxidation-associated conditions and diseases like inflammation or cancer. This study aimed to assess the bioactive potential of lipidic extracts obtained from *Gloeothece* sp.–using Generally Recognized as Safe (GRAS) solvents like ethanol, acetone, hexane:isopropanol (3:2) (HI) and ethyl lactate. The bioactive potential of extracts was assessed in terms of antioxidant (ABTS^•+^, DPPH^•^, ^•^NO and O_2_^•^assays), anti-inflammatory (HRBC membrane stabilization and Cox-2 screening assay), and antitumor capacity (death by TUNEL, and anti-proliferative by BrdU incorporation assay in AGS cancer cells); while its composition was characterized in terms of carotenoids and fatty acids, by HPLC-DAD and GC-FID methods, respectively. Results revealed a chemopreventive potential of the HI extract owing to its ability to: (I) scavenge ^-^NO^•^ radical (IC_50_, 1258 ± 0.353 µg·mL^−1^); (II) inhibit 50% of COX-2 expression at 130.2 ± 7.4 µg·mL^−1^; (III) protect 61.6 ± 9.2% of lysosomes from heat damage, and (IV) induce AGS cell death by 4.2-fold and avoid its proliferation up to 40% in a concentration of 23.2 ± 1.9 µg·mL^−1^. Hence, *Gloeothece* sp. extracts, namely HI, were revealed to have the potential to be used for nutraceutical purposes.

## 1. Introduction

The first reports on cyanobacteria date back to the time of Aztecs who used *Spirulina* (*Arthrospira platensis*, *A. maxima*) as food [1]. Nowadays the potential application of cyanobacteria in our daily lives has been well documented. Such microscopic organisms are indeed a universal source of a vast array of chemical products with applications in the feed, food, nutritional, cosmetic, and pharmaceutical industries [1,2,3]. The last decades have witnessed the massive development in the production of cyanobacteria through the improvement of processing methods, with particular emphasis on the extraction of high-value compounds to be used as nutraceuticals and pharmaceuticals [1,4].

Nevertheless, the exploitation of prokaryotic and eukaryotic microalgae is restricted to a few strains and most species remain largely unexplored. So far, till 2019, 260 families of bioactive compounds were identified in cyanobacteria with a wide range of applications, e.g., agriculture, pharmacology, cosmetology, or in the food industry; belonging to 10 different classes: alkaloids, depsipeptides, lipopeptides, macrolides/lactones, peptides, terpenes, polysaccharides, lipids, polyketides, and others [5]. Additionally, 14 major activities have been listed from the literature, among them are cytotoxicity, anti-inflammatory, and antioxidant, activities, at which bioactivities are particularly attributed to carotenoids, chlorophylls, mycosporine-like amino acids, and phycocyanins [5].

Extensive research efforts during the last decades revealed that continued oxidative stress may activate mechanisms that lead to chronic inflammation—which, in turn, could mediate chronic diseases like cancer. Oxidative stress occurs due to an imbalance between the production of free radicals, such as reactive oxygen species (ROS), and their elimination by natural protective mechanisms, such as antioxidants molecules. This imbalance may lead to injury of vital biomolecules, cells, and eventually the whole organism [6]. Therefore, the search for antioxidants or radical scavengers able to neutralize the harmful effects of oxidative stress has been in order, as they would prevent or treat inflammation or cancer [7,8].

Cancer is nowadays the 6th leading single cause of death worldwide [9]. This disease occurs due to an imbalance between the rate of cell proliferation and apoptosis; thus, an ideal therapy would be based on the ability to restore this balance, by either reducing cancer cell growth and/or promoting cancer cell death [10]. Gastric cancer ranks as the 5th most common type of cancer, and is the 3rd in cancer-related death [11]; its development has been frequently associated with severe inflammation caused by bacterium *Helicobacter pylori* [12].

**Figure 1:**
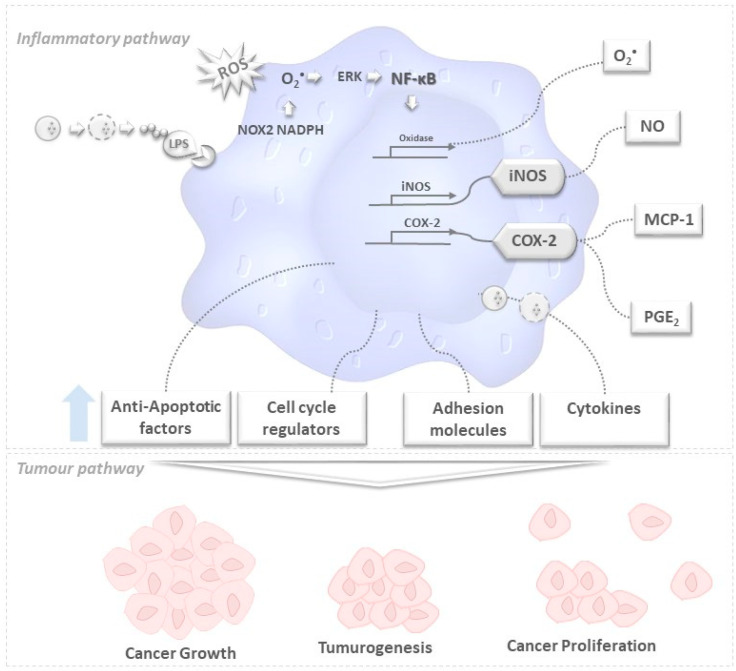
Brief schematic representation of how oxidative stress, inflammation, and cancer development may be correlated. After the lipopolysaccharide (LPS) inflammatory activation pathway in a macrophage cell, secretory lysosomes (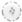) secrete or degrade inflammatory cytokines in regulating cytokines released by immune cells through a feedback mechanism. Phosphorylation of NF-κB activates several enzymes, e.g., cyclooxygenase(COX-2), oxidase, and iNOS, thus inducing the release of prostaglandins (PGE2), O_2_^•−^ and other molecules like anti-apoptotic factors, cell cycle regulators, adhesion molecules that are likely to be related to tumorogenesis, cancer cell growth and proliferation. The unbalanced increase of the former may lead to tumorogenesis and (among other events) cancer growth and proliferation.

It should be emphasized that it was found long ago that oxidative stress, chronic inflammation, and cancer development are closely related, particularly in what concerns their activation pathways—which entail the production of several inflammatory signaling molecules, like prostaglandins (PGs) as well as oxygen- and nitrogen-derived free radicals, as schematized in Figure 1 [7], a key characteristic of tumor promoters is their ability to recruit inflammatory cells and to stimulate them to generate ROS [7,13]. On the other hand, ROS are usually generated during mitochondrial metabolism and play an important role in cell signaling and homeostasis. ROS such as NO^•^, are produced during the inflammatory process [14] in response to inflammatory stimuli (e.g., cytokines or pathogens)—and some cases of deregulated inflammatory responses; thus may accordingly promote a state of chronic oxidative stress and inflammation [15].

The triggering of the inflammatory pathway by lipopolysaccharides (LPS) causes rapid activation of NOX2 and NADPH oxidase, and release of internal O_2_^•−^. This radical triggers, in turn, NF-κB phosphorylation, by activating several enzymes—namely cyclooxygenase 2 (COX-2), and iNOS which induce the release of PGE2, free radicals like O_2_^•−^ and NO, and the chemokine MCP-1. Other activation products of NF-κB include anti-apoptotic factors, cell cycle regulators, and adhesion molecules—which may be related to cancer cells’ survival, proliferation, adhesion, invasion and metastasis, and angiogenesis [16]. Of note, the release of such mediators, like cytokines, may be regulated by secretory lysosomes. Indeed, secretory lysosomes can secrete or degrade inflammatory cytokines in the regulation of cytokine release, thus positively and negatively regulating the inflammation, having a feedback mechanism to adjust the balance of the inflammatory response in cells and organelles. Furthermore, involvement of a lysosomal membrane protein in the activation of NF-κB and other pathways suggests that the lysosomal compartments may play a central role in the inflammatory signaling network—and accordingly, provide a theoretical basis for the development of anti-inflammatory drug combinations consisting of a lysosomal inhibitor [17], see Figure 1.

Another common strategy followed in the formulation of anti-inflammatory agents is based on suppressing of production of inflammatory mediators, such as COX-2 inhibitors, that interfere with the initiation and progression of inflammation-associated diseases [18]. PGs were found in several kinds of tumors, like gastric cancer [19] or colon adenocarcinoma [20]; causing tumorigenic effects, such as stimulation of cell growth and angiogenesis, inhibition of apoptosis, and suppression of the immune system. Several studies also indicate that COX-2 inhibitors can reduce the risk of development of colon, lung, or skin cancer [21,22,23], and namely improve therapeutic effects on human cancers in combination with chemotherapeutic [24].

In practice, the synthetic drugs used to treat these disorders may bring about severe side effects; hence is important to find compounds from biological sources, such as cyanobacteria, lacking adverse effects [25]. Carotenoids and PUFA from microalgal sources have indeed been claimed to have anti-cancer and anti-inflammatory properties, having sometimes an antioxidant-based mechanism of action [26,27,28]. Some of them have even been proposed for the treatment and prevention of such chronic diseases [29,30]. Epidemiological studies suggest that carotenoids can prevent free radical-dependent oxidation of LDL, cholesterol, proteins or DNA, by capturing free radicals and thus reducing stress induced by ROS [31]. Furthermore, PUFA, namely n-3 PUFA, was described to hold antioxidant and anti-inflammatory effects [32,33,34].

In the particular case of cancer, some strategies of chemoprevention can be accomplished by incorporating antioxidant compounds in the diet, which would block or delay cancer development, either in the initial phase of carcinogenesis or at the stage of progression of neoplastic cells to cancer [35]. A clear example is β-carotene, which protective effect against cancer was intimately associated with its antioxidant role [2] and COX-2 suppression abilities [36]. Moreover, the potential of microalgal lipidic components as chemopreventive agents was observed in colon, skin, and stomach cancer [2]. Also, other carotenoids such as violaxanthin, zeaxanthin, lutein, and fucoxanthin, or ethanol-based carotenoids-extracts, isolated from microalgae, exhibited antiproliferative activity against different cancer cells [27,35,37,38,39,40].

For this study, a scarcely studied prokaryotic colonial microalga was selected, *Gloeothece* sp., with promising bioactive lipidic composition [41]. This study aimed to exploit the bioactive potential of its lipid extracts, as a new source of antioxidant, anti-inflammatory, and antitumor compounds—thus forecasting a possible application in the food and nutraceutical industry. Hence, GRAS (Generally Recognized as Safe) solvents—ethanol, acetone, ethyl lactate, and a mixture (3:2) of hexane/isopropanol, were selected to extract lipidic bioactive compounds from *Gloeothece* sp. [42,43].

## 2. Results

### 2.1. Biochemical Composition of Extracts

**Figure 2:**
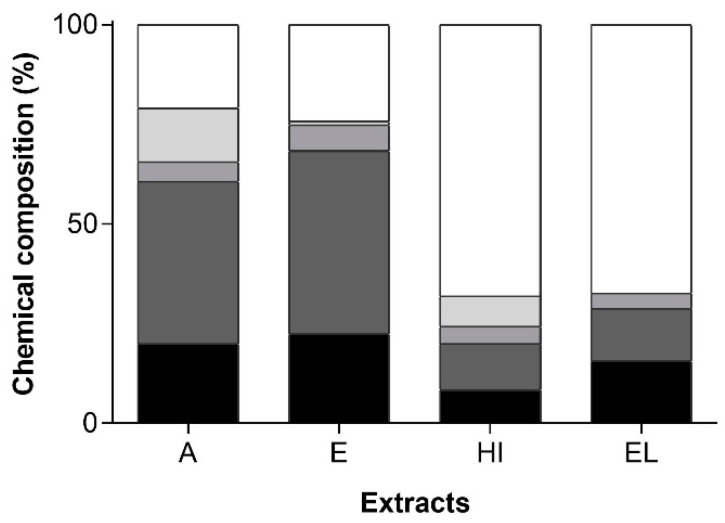
*Gloeothece* sp. extract’s composition (m_C_/m_E_, %) in terms of 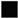 MUFA, 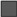 PUFA, 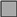 carotenoids, 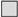 phenolic compounds, and other unidentified compounds, obtained with acetone (A), ethanol (E), hexane:isopropanol (1:1, *v*/*v*) (HI) and ethyl lactate (EL).

*Gloeothece* sp. extracts may have the potential of application in the nutraceutical industry, due to their content in bioactive compounds as carotenoids, polyunsaturated fatty acids (PUFA), or phenolic compounds. First, a crude characterization of extracts composition in terms of each family of compounds (m_C_/m_E_, %) was done, as depicted in Figure 2.

It can be observed that A and E extracts are mainly composed of fatty acids, ca. 60 and 66%, respectively, most of them PUFA (more than 40%). Extract A also exhibited the highest percent composition in phenolic compounds (13%, m_C_/m_E_), followed by HI extract (ca. 8%, m_C_/m_E_). The contents of carotenoids were ca. 4% in all extracts, except for E, which reaches 6.5%.

A detailed fatty acids composition, available in Table 1, reveals different profiles in monounsaturated (MUFA) and polyunsaturated (PUFA) fatty acids, either in terms of concentration (μg_Fatty Acid_·mg_Extract_^−1^) and content (%, m_Fatty Acid_/m_Total Fatty Acid_).

Concerning the MUFA C18:1 n9 c+t (oleic acid, OA), this is the one present in higher content and the 3rd in terms of all fatty acids. Its content in all extracts ranges between 14.4 (E) and 17.4% (EL), having a higher concentration in extract A, 53.796 ± 2.918 μg_Fatty Acid_·mg_Extract_^−1^—i.e., approximately half of concentration in E, and one quarter in HI and EL.

In terms of PUFA, E and A exhibited a higher content, 40.7 and 46.0% (m_FA_/m_TFA)_, respectively. In another way, HI and EL accounted for 80 and 71.3 (%, m_FA_/m_TFA_), respectively, in saturated fatty acids (data not shown).

Among PUFA, C18:3 n3 (α-linolenic acid, ALA) attained the highest content in all extracts, between 23.4 (E) and 26.4 (EL) % (m_FA_/m_TFA_); but with a higher concentration in A (96.765 ± 5.713 μg_FA_·mg_E_^−1^); followed by E (37.233 ± 0.685 μg_FA_·mg_E_^−1^), HI (13.216 ± 0.225 μg_FA_·mg_E_^−1^), and EL (4.575 ± 1.437 μg_FA_·mg_E_^−1^). In other way, the PUFA C18:2 n6 t (linoleic acid, LA) attained the highest concentration, 6.240 ± 1.510 μg_FA_·mg_E_^−1^, in the EL extracts. Note that conjugated linoleic acid (CLA, C18:2 n6 t + C18:2 n6 c), also in high content (%, m_FA_/m_TFA_) and concentration (μg_FA_·mg_E_^−1^) in E, (15.5%, 60.695 μg_FA_·mg_E_^−1^), followed by EL (6.6%, 6.577 μg_FA_·mg_E_^−1^), HI (5%, 11.991 μg_FA_·mg_E_^−1^), and A (1.5%, 24.648 μg_FA_·mg_E_^−1^). Furthermore, C20:5 n3, (eicosapentaenoic acid, EPA) was detected in EL and HI extracts, in concentration of 0.462 ± 0.071 and 0.283 ± 0.105 μg_FA_·mg_E_^−1^, respectively.

**Figure 3:**
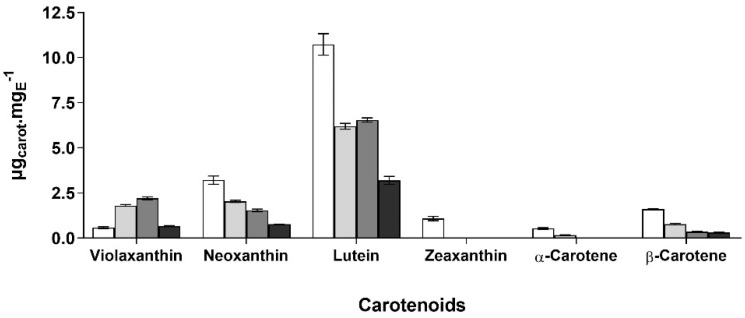
Carotenoid profile and content (µg_carotenoids_·mg_Extract_^−1^) in each *Gloeothece* sp. extract, 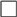 Acetone (A), 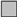 Ethanol (E) 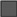 Hexane:Isopropanol (3:2) (HI) and 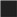 Ethyl Lactate (EL) extracts.

Observing the carotenoid profile and concentration (see Figure 3), extract A—besides having the highest concentration in total carotenoids, contains a quite different profile from the others, while E and HI profiles appeared to be similar. In another way, EL contains the fewest carotenoids and lowest content. Lutein is the most abundant carotenoid in all extracts, being ca. 35% more concentrated in A (10.73 ± 0.59 µg_carot_·mg_E_^−1^) than in E and HI, and 69% more than in EL, 3.19 ± 0.22 µg_carot_·mg_E_^−1^. Neoxanthin is the second most abundant xanthophyll, with 3.21 ± 0.23 µg_carot_·mg_E_^−1^ in A, i.e., 1.5-fold that of E, 2.1-fold of HI, and 4.1-fold of EL. Moreover, A is the only extract than contains zeaxanthin 1.07 ± 0.12 µg_carot_·mg_E_^−1^, and the highest concentration of α-carotene, i.e., 0.53 ± 0.04 µg_carot_·mg_E_^−1^, and β-carotene, i.e., 1.60 ± 0.03 µg_carot_·mg_E_^−1^.

### 2.2. Antioxidant Capacity of Lipidic Extracts

The extracts were tested for their total antioxidant capacity (via ABTS^•+^ and DPPH^•^ methods), and specific radical antioxidant capacity for radicals O_2_^•−^ and ^•^NO.

As observed in Table 2, all extracts exhibited total antioxidant capacity—although in some assays the IC_50_ values could not be estimated within the range of concentrations tested, such as O_2_^•−^ assay.

No significant differences were found between E and A extracts (*p* < 0.05) in ABTS^•+^ assay, and A extract exhibited the lowest IC_50_ in DPPH^•^ and ^•^NO^−^ assays. Although the IC_50_ for EL extract at ^•^NO^−^ assay could not be calculated in the range of concentrations tested, it was revealed to have antioxidant capacity.

### 2.3. Antitumoral Features of Lipidic Extracts

Among the available cancer adenocarcinoma cell lines, AGS highlights as being the gastric line most used in vitro study models [44]. Hence, antitumor capacities of all extracts were evaluated through different assays, using AGS cell line as a model. First, the cancer cell viability was evaluated by Sulforhodamine B assay, and IC_50_ was determined for each extract. The IC_50_ values of each extract were then used to determine whether the extracts were able to promote cell death via TUNEL assay; and whether the extracts were able to inhibit cancer cell proliferation, via cell proliferation BrdU assay.

#### 2.3.1. Evaluation of Cancer Cell Viability by Sulforhodamine B Assay

Sulforhodamine B assay (SRB) uses the protein-binding dye SRB to indirectly assess cell growth [45,46].

Despite DMSO being widely described to be cytotoxic depending on its concentration—yet, it was used to suspend extracts at low and non-cytotoxic concentrations. DMSO was thus titrated in these cell lines and, it was found that a concentration of 0.25% (*v*/*v*) was innocuous to AGS cells (data not shown).

For each extract, a dose-response curve was established, allowing determination of the extract’s concentration causing a cell growth inhibition of 50%, as shown in Table 2.

From the results calculated in Table 2, HI extract outstands for its lowest IC_50_ values, reaching values 5- to 10-fold lower when compared to the other extracts. IC50 values determined for each extract were then used to perform the cancer cell death and proliferation assays.

#### 2.3.2. Evaluation of Cancer Cell Death via TUNEL Assay

**Figure 4:**
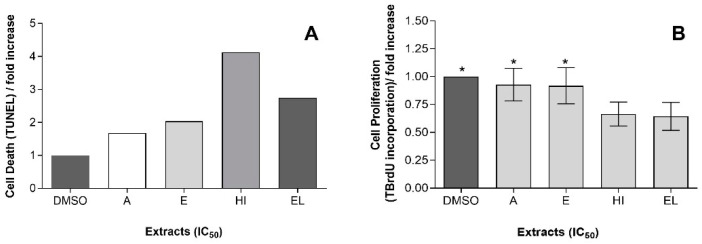
Antitumoral features of *Gloeothece* sp. lipidic extracts (**A**) AGS cell death, quantified by fold increase 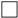 Acetone (A), 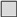 Ethanol (E), 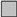 hexane:isopropanol (3:2) (HI) and 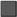 Ethylic lactate (EL) extracts; and, (**B**) AGS cell proliferation, quantified by fold increase by Acetone (A), Ethanol (E), hexane:isopropanol (3:2) (HI) and Ethylic lactate (EL) extracts, using DMSO as a negative control. Bars with a common character are significantly not different (*p* < 0.05) from the DMSO control.

TUNEL is a common method for detecting DNA fragmentation that may result from cell death, either by apoptosis or necrosis [47]. Induction of DNA fragmentation in AGS cells, treated with the different extracts, at their IC_50_ by 48 h of treatment, was examined using TUNEL. The results produced (Figure 4) show that treatment with all four extracts results in a significantly increased cell death (*p* ˂ 0.05), yet a stronger effect was observed for HI extract—which increased AGS cells death by c.a. of 4-fold.

#### 2.3.3. Evaluation of Cancer Cell Proliferation

Assessment of cell proliferation by BrdU assay is based on the incorporation of BrdU into their replicating DNA, which can further be detected by immunofluorescence. For a quantitative approach, samples were analyzed by flow cytometry. Results revealed an anti-proliferative effect of the HI and EL extracts upon AGS, via 40% of inhibition of proliferation in ca., while cells treated with the E or A extracts behaved no differently from the negative control with DMSO (Figure 4), i.e., exhibited no antiproliferative effect.

### 2.4. Anti-Inflammatory Potential of Lipidic Extracts

The mechanism of inflammation can be partially triggered via the release of ROS, from activated neutrophils and macrophages, thus leading to damage in macromolecules causing, namely, lipid peroxidation of membranes. ROS spread inflammation by stimulating the release of cytokines, regulated by lysosomes, which in turn stimulate the recruitment of additional neutrophils and macrophages. Lysosome structure conveys a physical and functional interface among cell organelles, as it plays a role in negative or positive modulation of the production of inflammatory cytokines [17,48]. Furthermore, free radicals are mediators that induce or sustain inflammatory processes; hence their neutralization by antioxidants and radical scavengers are fundamental to reducing inflammation [49]. In this context, extracts from *Gloeothece* sp. were screened for their potential anti-inflammatory features, by resorting to two different assays, one reflecting the stabilization of extracts on Human red blood cell (HRBC) membrane induced by heat, and another that ascertains the capacity of such extracts to inhibit the human enzyme COX-2.

#### 2.4.1. Human Red Blood Cell (HRBC) Membrane Stabilization Assay

This assay allows the characterization of the capacity of *Gloeothece* sp. extracts to protect erythrocytes from hemolysis when heat is supplied. Since the erythrocyte membrane is quite similar to the lysosomal one, indirectly is possible to conclude if any *Gloeothece* sp. extract holds any capacity in the stabilization of lysosomal membranes [50], and so, if they have the potential to be used as a non-steroidal drug—the common anti-inflammatory drug that inhibits lysosomal enzymes or stabilizes their membrane.

Results show that the HI 3:2 (*v*/*v*) extract is the most promising as it exhibits a protection capacity of 61.6 ± 9.6%; nonetheless, EL extract also appears to hold some potential in protecting HRBC membranes. Conversely, the E and A extracts did not show significant protective capacity (see Table 3).

#### 2.4.2. Cox Human Inhibitory Assay

Cyclooxygenases (COXs) catalyze reactions that lead to the formation of pro-inflammatory prostaglandins (PG), thromboxanes, and prostacyclins. Hence, the ability of extracts to inhibit the conversion of AA to Prostaglandin H2 (PGH2) via inhibition of COX-2 was determined. All concentrations tested exhibit anti-inflammatory activity in vitro, by inhibiting PG production in a dose-dependent manner. However, the extracts exhibited different behaviors within the range of concentrations tested, data not shown.

While A and EL at lower extract concentration induces a higher inhibition, a linear percent of inhibition is of E concentration was observed, whereas a non-significantly percentage of inhibition variation was detected with HI concentration. In terms of total inhibition capacity of COX-2 enzymatic activity, one notices that A, E, and HI performed equally well beyond 50% with no significant differences between them (*p* ˂ 0.05); however, the corresponding IC50 values (see Table 3) revealed that A and HI extracts attained the lowest values, without significant differences (*p* ˂ 0.05).

### 2.5. Cytotoxicity

**Figure 5:**
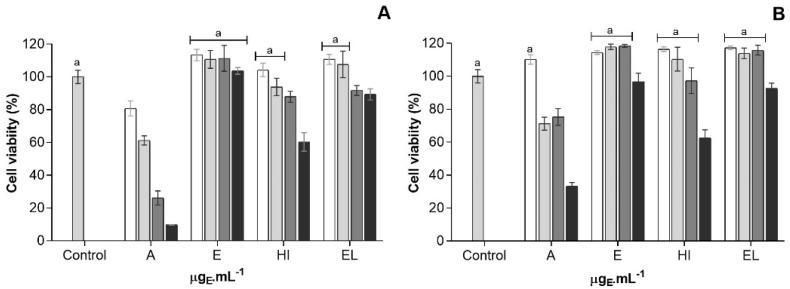
Cytotoxicity evaluation of Acetone (A), Ethanol (E), Hexane:Isopropanol (3:2) (HI) and Ethyl Lactate (EL) *Gloeothece* sp. extracts, against HCMEC cell line, tested at 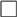 50, 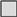 100, 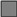 200 and 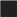 300 μg·mL^−1^, by 24 h (**A**) and 48 h (**B**). Bars marked with the same letter in the same superscript have no significant difference relative to the control (*p* < 0.05).

For a putative application of *Gloeothece* sp. extracts as a nutraceutical ingredient, it is mandatory that extracts do not exhibit any cytotoxicity to non-cancer cells. Therefore, cytotoxicity effects upon HCMEC cells were assessed after 24 h (see Figure 5A) and 48 h (see Figure 5B), using DMSO 1% as a negative control. Results show that A extract is cytotoxic, although its cytotoxicity decreases after 48 h. However, promising results were observed concerning the E extract, since there was no evidence of cytotoxicity at all concentrations tested. On the other hand, both HI and EL extracts were not lethal up to 100 μg·mL^−1^; the highest concentrations tested were toxic, although toxicity decreases with time.

## 3. Discussion

Drugs commonly used to treat inflammation and cancer raise severe side effects, such as toxicity and decreased life quality [51,52]. In this regard, this work aimed at making a preliminary test of *Gloeothece* sp. extracts to be eventually used as a natural source in nutraceuticals, and/or as a potential chemopreventive agent—based on the composition in carotenoids and PUFA, coupled with antioxidant, antitumoral, and anti-inflammatory features. Pearson correlations were calculated (data not shown) between composition (carotenoids and PUFA) and bioactive features, however possible synergetic effects among the molecules, that were not possible to measure, may contribute to its bioactive potential. Hence, these features will be discussed separately, and then in an integrated manner.

### 3.1. Antioxidant Capacity of Lipidic Crude Extracts

The antioxidant capacity of cyanobacterial carotenoids is well established—particularly concerning lutein and β-carotene [27,29,30,53], and long-chain fatty acids such ω3 PUFA [30,32]. Analyzing the extract contents in PUFA (see Table 1), carotenoids (see Figure 2) and, it results of total antioxidant capacity, it is possible to correlate extract concentration of carotenoids and PUFA with antioxidant bioactivity—at which A extract, stands out due to its lowest IC_50_ values at all antioxidant assays. As observed previously, lutein probably contributes the most to said bioactivity, owing to its higher concentration [54]. However, other carotenoids (e.g., β-carotene and neoxanthin) should not be overlooked owing to their concentrations, as well as such PUFA as 18:1 n9, 18:2 n6, and 18:3 n3 based on the IC_50_ values of *Gloeothece* sp. extracts (A > E > HI > EL). Particularly, a correlation was found with C18:2 n6 (*r* = 1, *p* < 0.083).

Concerning the specific radical’s scavenger capacity, results reveal the same trend, particularly in NO^•^ assay, in which the lowest IC_50_ was again observed in the A extract. The high concentration of total carotenoids and PUFA, namely lutein and C18:2 n6, may account for their important antioxidant role (*r* = 1, *p* < 0.083), as reported before [55,56,57].

Although the IC_50_ values for the O_2_^•−^ assay could not be found at the tested concentrations, some scavenging effects were detected at E and EL extracts—data not shown.

Hence, owing to the antioxidant scavenging capacity of A and E extracts against NO^•^ and O_2_^•−^ radicals in vitro, a similar capacity is expected in vivo—with a preventive role of chronic inflammatory diseases, cancer, or neurodegenerative disorders [58,59].

### 3.2. Antitumoral Features of Cyanobacterial Extracts

Unlike observed with antioxidant capacity, the most promising extracts, in terms of inducing AGS cell death and cell proliferation, are HI and EL extracts; where it cannot be established a clear correlation of antitumor capacity and high content in carotenoids and fatty acids.

Despite a possible interaction of all extracts’ compounds, some evidence relate such bioactivities with some compounds identified in *Gloeothece* sp. extracts, such as phenolic compounds. Although these compounds have not been characterized, the content in aromatic compounds is described to exert effects in bioactivities, particularly in antitumor and anti-inflammatory agents [60].

From a nutraceutical point of view, dietary supplementation of β-carotene in animal models of colon carcinogenesis has revealed anticancer capacities for that compound [61], as well as growth-inhibitory and pro-apoptotic effects in human colon cancer cell lines [36]. It has also been demonstrated that such chemopreventive activity is dose-dependent, a high dose proving to be harmful and likely to have a proliferative effect upon some cancer cells lines [1]; this may explain why the HI and EL extracts, characterized by the lowest levels of β-carotene and lowest IC50 values, exhibited the best results upon cancer cell death and proliferation. Additionally, such xanthophylls, violaxanthin have been found to possess antiproliferative activity against different cancer cells [35], and in fact, HI extract exhibited the highest level of violaxanthin.

Some PUFA, particularly ω-3, have been reported to possess in vitro and in vivo anticancer effects, via modulation of tumor growth or increase of cell death rate [62,63], this is the particular case of EPA, able to inhibit some cancer cell lines proliferation in a dose-dependent and time-dependent manner [62]. However, particular attention should go to LA. Studies reveal that treatment of AGS and MKN cells with linoleic acid (C18:2n6), in which EL extract has the higher content, led to an increase in a proapoptotic protein expression and a decrease of an anti-apoptotic protein expression, as well as inhibits the production of PGE2 and activity of telomerase by suppressing COX-2 and hTERT expression, in a dose-dependent manner [64,65], which may be in line with our results in AGS cell death. Indeed, in our study, a correlation was found between cell death and C18:2n6 content (*r* = 1, *p* < 0.083).

It should be noted that the antitumoral IC_50_ value for the HI extract (23.2 ± 1.9 µg·mL^−1^) is lower than other hexanoic extracts reported before for human colon carcinoma cell line (HCT116), for example for *Chlorella ellipsoidea* and *C. vulgaris* which IC_50_ value was ca. 41µg·mL^−1^ and equivalent to the one obtained with pure lutein (21.02 ± 0.85 µg·mL^−1^) [39]. Also, correlation was found for AGS cell proliferation and content of C18:1 n9, C18:2 n6, C18:3 n3 and β-carotene contents (*r* = 1, *p* < 0.083).

### 3.3. Anti-Inflammatory Potential of Lipidic Crude Extracts

The anti-inflammatory potential of *Gloeothece* sp. extracts was assessed by two assays. In the HRBC assay, HI extracts stood out in terms of inhibition capacity of 61%; hence, this HI extract may potentially stabilize cell membrane and thus prevent stress-induced decay, as well as stabilize the lysosomal membrane. This feature is crucial in the prevention of an anti-inflammatory response induced by the release of lysosomal constituents, which cause further tissue inflammation and damage upon extracellular release [50].

As seen before, the ability to inactivate COX-2 is indicative of the potential of an extract to be used as an anti-inflammatory drug. All extracts of *Gloeothece* sp. exhibited that ability, some of them having a dose-dependent response, like E extracts. However, extract A exhibited the best performance at a concentration of 75 µg·mL^−1^, inhibiting in ca. 57% of COX-2 enzymatic activity; however, the possible application of A extracts use must be discarded due to its cytotoxicity to HCMEC cells. Nonetheless, HI extract follows as most promising due to ca. 48% of inactivation capacity and with no cytotoxicity associated.

A number of anti-inflammatory molecules obtained from microalgae have been shown to display high antioxidant capacity, that is in the composition of A and HI, such as β-carotene, lutein, zeaxanthin, and ω3 PUFA [66]. Some of the anti-inflammatory ability could be attributed to violaxanthin. This xanthophyll isolated from *C. ellipsoidea* showed anti-inflammatory activity when it was tested on LPS-stimulated RAW 264.7 mouse macrophages, by inhibiting NF-κB activation and NO and prostaglandin E2 (PGE2) production [67].

### 3.4. Potential of Application of Gloeothece sp. Extracts

Chemoprevention consists of the use of pharmaceutical drugs, or nutritional supplements to reduce the risk of developing or having a recurrence of cancer. Several in vitro and animal studies showed the chemopreventive properties of a few metabolites from microalgae (e.g., carotenoids, fatty acids, polysaccharides, and proteins), namely against colon and skin cancer [2].

Performance recorded for *Gloeothece* sp. extracts, particularly the A and HI shows that they are a promising source in the eventual formulation of some nutraceutical products bearing antioxidant, anticancer, and anti-inflammatory capacities. But despite the notable antioxidant features of the A extract, particularly its ability to inhibit the radical NO^•^, its potential application as a nutraceutical is limited due to its cytotoxicity.

**Figure 6:**
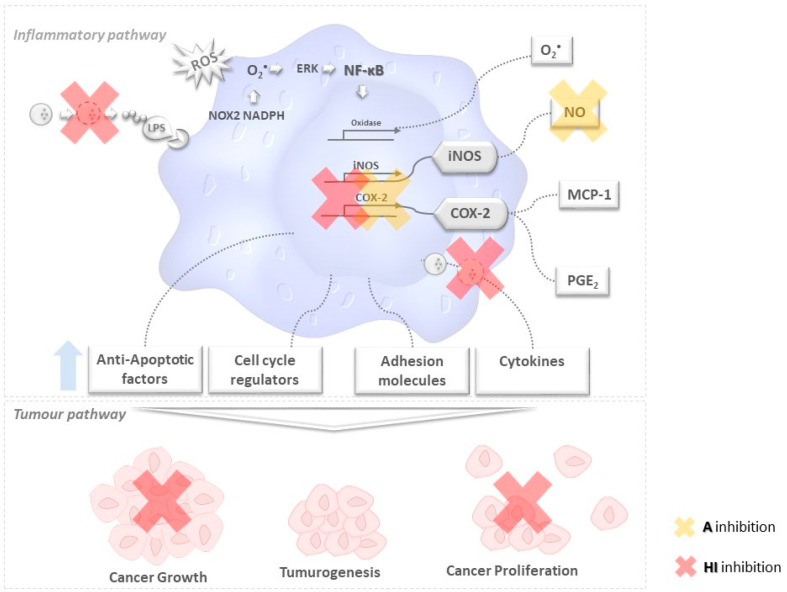
Schematic representation of how the HI (red cross) and A (yellow cross) extracts may modulate oxidative stress, inflammation, and cancer development. The HI extract protects membranes of secretory lysosomes, thus avoiding the release of inflammatory cytokines and consequent feedback mechanism. The phosphorylation of NF-κB is activated. A is able to reduce the produced NO radicals. HI and A are able to suppress cyclooxygenase (COX-2), and subsequent release of prostaglandins (PGE2), as well as anti-apoptotic factors, cell cycle regulators, adhesion molecules related to tumorogenesis, cancer cell growth, and proliferation. HI extract is able to inhibit cancer-related events such as cancer growth and proliferation.

Experimental and epidemiological evidence reported before suggests that anti-inflammatory drugs may also decrease the incidence of some types of cancer, as well as tumor burden and volume [68,69]. An attempt to provide a global overview of the potential of action of HI and A extracts is conveyed by Figure 6.

Hence, the HI extracts of *Gloethece* sp. appeared to be the most promising as a chemo-preventive agent in the nutraceutical industry because of their features as (1) antioxidant namely high total antioxidant capacity and scavenging capacity against ^-^NO^•^ radical; (2) antitumor induction of cell death upon AGS cells, along with anti-proliferative effects; and (3) anti-inflammatory, namely inability to inhibit COX-2 expression while protecting lysosomes.

## 4. Materials and Methods

### 4.1. Microorganism Source and Biomass Production

*Gloeothece* sp. (ATCC 27152) was purchased from ATCC—American Type Culture Collection (USA), and kept at 25 °C, using Blue Green (BG11) as culture medium [70]. For biomass production, in 4 L batch culture, first, a pre-inoculum, with an initial optical density of 0.1 at 680 nm, was cultivated for 10 days in 800 mL of BG11 medium, buffered at pH 8 with Tri-(hydroxymethyl)-aminomethane hydrochloride (Tris-HCl)—ensuring that the microorganism was at the exponential growth phase at the time of inoculation for biomass production. Hence, biomass production was started with an initial optical density of 0.1 in BG11 medium buffered at pH 8 and was produced for 14 days under a continuous illumination with fluorescent BlOLUX lamps, with an intensity of 150 µmol_photon_·m^−2^·s^−1^, and air bubbling at a flow rate of 0.5 L·min^−1^. Biomass was then collected by centrifugation at 18× *g* for 10 min, the supernatant was rejected and pellet freeze-dried, and stored under gaseous nitrogen until analyses were performed.

### 4.2. Extract Preparation

Extracts from *Gloeothece* sp. were obtained from 200 mg of lyophilized biomass, using four alternative food-grade solvents (Fisher Chemical, New Hampshire, EUA): ethanol (E), acetone (A), a mixture (3:2) of hexane/isopropanol (HI), and ethyl lactate (EL), as previously tested [41].

### 4.3. Chemical Characterization of Extracts

Fatty acids and carotenoids are among the most widely known bioactive compounds found in microalgae, which possess a high interest in the nutraceutical and pharmaceutical markets; hence, solvent extracts were evaporated and residue composition was determined for each *Gloeothece* sp. extract, as detailed below.

#### 4.3.1. Profile and Content of Polyunsaturated Fatty Acids

The weighted residue was submitted to direct transesterification to produce fatty acid methyl esters according to the acidic method described by Lepage and Roy [71], after modifications introduced by Cohen et al. [72] using acetyl chloride (Sigma-Aldrich, St. Louis, MO, USA) as catalyst. The internal standard used was heptadecanoic (C17:0, Sigma-Aldrich, St. Louis, MO, USA) acid and esters were analyzed in a Varian Chrompack CP-3800 gas chromatograph (GC), using a flame ionization detector, and quantified with the software Varian Star Chromatography Workstation (USA, Version 5.50). Helium was employed as the carrier gas in splitless mode and the silica CP-WAX 52 CB (Agilent) column was used. The injector and detector were maintained at 260 and 280 °C, respectively, and the oven heating program was the same as described before [42]. To identify PUFA, chromatographic grade standards of fatty acids were used in methyl ester form CRM47885 (Supelco, St. Louis, MO, USA). Concentrations of each polyunsaturated fatty acid (PUFAs) were determined and mean values were used as a datum point.

#### 4.3.2. Profile and Content of Carotenoids

To determine the content in carotenoids of the extracts, high-performance liquid chromatography (HPLC) was applied as an analytical technique as detailed before [54]. The residue was weighed and resuspended in acetone: acetonitrile (9:1); 8-β-apo-carotenol (Sigma-Aldrich, St. Louis, MO, EUA) was used as internal standard. Standards were purchased from CarotNature, Lutein (No. 0133, Xanthophyll, (3R,3′R,6′R)-β,ɛ-Carotene-3,3′-diol with 5% Zeaxanthin with 96% purity), β-carotene (No. 0003, β, β-carotene) with 96% purity) and β-apo-carotenol (No. 0482, 8′-Apo-β-caroten-8′-al) with 97% purity). The elution times of the chromatographic standards were: 14.4 min for lutein and 34.4 min for β-carotene. Identification was by comparison of retention times and UV–visible photo-diode array spectra, following the procedure by Guedes [54].

### 4.4. Antioxidant Effects of Lipidic Extracts

The antioxidant capacity of each extract was evaluated via four spectrophotometric assays: two assessed total antioxidant capacity (ABTS^+•^, DPPH^•^); while the other two were more specific for two biological radicals, superoxide (O_2_^•−^) and nitric oxide (^•^NO^−^)—with the later be known to be correlated with inflammation processes.

A positive control, Trolox, was used to validate the antioxidant capacity of extracts and putatively establish a calibration curve but comparing the antioxidant capacity of the extracts, their IC_50_ values were established. A dilution series was accordingly prepared for each extract, with concentrations ranging from 0.440 to 7 mg·mL^−1^—for ethanol, acetone, and HI extracts, and from 1.5 to 24 mg·mL^−1^ for ethyl lactate extract, in Phosphate Buffered Saline (PBS) containing 5% of DMSO. Each antioxidant assay was performed in triplicate, as described in the following sub-sections.

#### 4.4.1. ABTS^+•^ Scavenging Capacity

The total antioxidant capacity was determined as the capacity to decrease the absorbance of blue/green chromophore 2,2′-Azino-bis (3-ethylbenzothiazoline-6-sulfonic acid) (ABTS^•+^) (Alfa Aesar, Massachusetts, US). Absorbance was accordingly determined at 734 nm, upon the reaction of the extract with ABTS^•+^ for 6 min—as previously optimized by Guedes et al. [54].

#### 4.4.2. DPPH^•^ Scavenging Capacity

The antioxidant capacity was determined, in triplicate, by reacting each extract with 2,2-diphenyl-1-picrylhydrazyl (DPPH^•^) (Sigma-Aldrich (St. Louis, MO, USA), after an incubation period of 30 min at room temperature in dark. The scavenging reaction was monitored at 515 nm, as implemented before by Amaro et al. [41].

#### 4.4.3. Superoxide Radical (O_2_^•^^−^) Scavenging Capacity

Superoxide radicals are generated by the NADH/PMS system. The extract antioxidant capacity was determined by monitoring the absorbance of the reaction mixture, at 560 nm and room temperature, for 2 min, as previously performed by Amaro et al. [41].

#### 4.4.4. Nitric Oxide Radical (^•^NO^−^) Scavenging Capacity

Each extract was incubated with sodium nitroprusside, for 60 min at room temperature, in the light. Griess reagent was added afterward, and the chromophore reaction was carried out in the dark for 10 min; absorbance was read at 562 nm [41].

### 4.5. Anticancer Effects of Gloeothece sp. Extract

#### 4.5.1. Cancer Cell Culture

Human gastric carcinoma cell line AGS CRL-1739 (obtained from ATCC, USA) derived from fragments of a tumor resected from a patient who had received no prior therapy, were maintained in RPMI1640 (Invitrogen, Thermo Fisher Scientific, Waltham, MA, USA) supplemented with 10% FBS (Lonza, Basel, Switzerland) and kept at 37 °C, in a humidified 5% CO_2_ incubator.

#### 4.5.2. Cancer Cell Viability Sulforhodamine B Assay

Solvents of each extract were evaporated by rotavapor and extracts resuspended with the minimum amount of dimethyl sulfoxide (DMSO) (AppliChem, Darmstadt, Germany), thus producing in concentrations of 130, 150, 120, and 450 mg·mL^−1^, for acetone, ethanol, HI and ethyl lactate extracts, respectively.

AGS cells in a concentration of 1 × 10^4^ were seeded in 96-wells plates and treated for 48 h with different concentrations of microalgal extracts (0 to 550 µg·mL^−1^ whenever possible) or DMSO (AppliChem, Darmstadt, Germany) as negative treatment control (0.05% *v*/*v*). As a positive control, DMSO 100%, was used to validate the antitumoral capacity of extracts. Then cells were fixed by the addition of 50 μL of cold 50% trichloroacetic acid (Merck Millipore, Kenilworth, NJ, USA) to each well, and incubating the plates at 4 °C for 1 h. Next the fixation step, the plates were washed three times with deionized water and dried at room temperature. The cells were then stained with 50 μL of 4% sulforhodamine B (SRB) (Sigma-Aldrich, St. Louis, MO, USA) in 1% acetic acid (Mallinckrodt Baker, Deventer, The Netherlands) for 30 min and then washed three times with deionized water. After the plates were dry, the cells were solubilized with 100 μL of 10 mM unbuffered Tris Base (Sigma-Aldrich, St. Louis, MO, USA), and the optical density at 510 nm was measured using the fluorimeter SynergyTM 4 Multi-Mode Microplate Reader (Biotek, Winooski, VT, USA). Results were plotted as dose-response curves, and the IC_50_ for each extract was found and expressed as µg_E_·mL^−1^.

#### 4.5.3. Cancer Cell Death TUNEL Assay

AGS cells were cultured in 6-well plates in a concentration of 7.5 × 10^5^, and treated for 48 h with the microalgal extracts at the IC_50_ found at the SRB assay, for 48 h. DMSO (AppliChem, Darmstadt, Germany) was used as a positive control treatment. Cells were washed and trypsinized and the pellet obtained was fixed in 3 mL of ice-cold methanol for 15 min. Then, cells were washed and resuspended in 500 μL of PBS. Incubation with TUNEL reaction mix (1:9:10 concerning the Dilution Buffer reagent, according to manufacture instructions—In Situ Cell Death Detection Kit Fluorescein, Roche, Mannheim, Germany) was done for 1 h, at 37 °C, in the dark. Then, data were acquired using a BD Accuri C6 flow cytometer (BD Biosciences, San Jose, CA, USA).

#### 4.5.4. Cancer Proliferative Assay

AGS cells were cultured in 6-well plates containing a concentration of 7.5 × 10^5^ and treated with the extracts at the IC_50_ found at the SRB assay, for 48 h, using DMSO (AppliChem, Darmstadt, Germany) as positive control treatment. 5-Bromo-2′-deoxyuridine (BrdU) (BrdU labeling and detection kit 1, Roche, Mannheim, Germany) was incorporated in the cell culture medium at the ratio of 1:1000, and underwent incubation for 1 h, at 37 °C. Straightaway the following incubation, the cells were harvested, washed with PBS, fixed in 1 mL of ice-cold methanol for 30 min, washed again, and resuspended in 500 μL of PBS. This was followed by the incubation with 1 mL of HCl 4 M (Mallinckrodt Baker, Deventer, The Netherlands), for 20 min, two washing steps with PBS, a blocking step (PBS containing 0.5% Tween 20 and 0.05% BSA), and finally 1 h incubation at room temperature with the primary antibody against BrdU (1:20, Bu20a, Dako, Glostrup, Denmark). Next, the cells were further washed with PBS and incubated with the secondary antibody labeled with FITC (1:200, polyclonal rabbit anti-mouse, Dako, Glostrup, Denmark), for 30 min at room temperature washed two times and resuspended in 500 μL of PBS. Data acquisition was performed with a BD Accuri C6 flow cytometer (BD Biosciences, San Jose, CA, USA).

### 4.6. Anti-Inflammatory Effects of Extracts

To assess the anti-inflammatory potential of the lipidic extracts, two assays were performed. The Human red blood cell (HRBC) membrane stabilization assay, induced by heat, was used first; it allowed to observe if any extract holds the potential to stabilize lysosomal membranes. The second assay is specific to a prostaglandin-endoperoxide synthase, human COX-2 enzymatic activity inhibition—and helps conclusion on whether any extract has the potential to be used as a non-steroidal anti-inflammatory agent. The study was conducted according to the guidelines of the Declaration of Helsinki, and ap-proved by the Institutional Ethics Committee of CIIMAR (protocol code 001/2020 and date of approval 8 June 2020).

#### 4.6.1. Human Red Blood Cell (HRBC) Membrane Stabilization Assay

Human fresh blood was collected intravenously to heparinized tubes, from a healthy volunteer that was not taking any non-steroidal anti-inflammatory drugs (NSAIDs) for 2 weeks before the experiment. Blood was centrifuged at 700× *g* for 10 min and supernatant (plasma) was removed. Hence human red blood cells (HRBC) were washed three times with an equal volume of isotonic PBS (10 mM sodium phosphate buffer(Alfa Aesar, Massachusetts, US) pH 7.4) and then reconstituted at 40% (*v*/*v*) suspension. Salicylic acid at 500 µg mL^−1^ was used for positive control and PBS with 20% of DMSO (AppliChem, Darmstadt, Germany) for negative control.

Each extract, prepared as explained in Section 2.2, at concentrations of 130, 150, 120, and 450 mg·mL^−1^, for A, E, HI, and EL, respectively, were resuspended in PBS containing 20% of DMSO, and then mixed in 1:1 (*v*/*v*) with a solution of HRBC in 2% in PBS. Samples were incubated at 56 °C for 20 min, cooled in tap water, and centrifuged at 700× *g* for 5 min, and the supernatant was collected. The absorbance of the supernatant was measured spectrophotometrically at 560 nm using a microplate reader (Thermofisher GO, New Hampshire, EUA) [73]. The percentage of inhibition was calculated for each extract as:

% inhibition = [(Abs_E_ − Abs_EB_) − Abs_C_]/Abs_C_ × 100 (1)

where Abs_E_ denotes supernatant absorbance after reaction with extract; Abs_EB_ denotes extract absorbance at 560 nm; and Abs_C_ denotes the control absorbance of PBS with 20% of DMSO.

#### 4.6.2. Cox Human Inhibitory Screening Assay

The anti-inflammatory potential of the extracts was assessed via an enzyme inhibitory assay—inhibition of COX-2 enzymatic activity, using the COX-2 Enzyme Activity Assay Kit (Cayman Chemical, Michigan, MI, US), according to the manufacturer’s instructions. Dried lipidic extracts were diluted in DMSO, and assayed at different concentrations—75, 125, and 250 µg·mL^−1^.

In this assay, arachidonic acid (AA) served as a substrate for the human recombinant COX-2 enzyme, thus leading to the production of prostaglandin. The assay measures PGF2α produced by SnCl2 reduction of COX-derived PGH2. The PGF2α levels produced in the presence versus absence of test products were quantified through an enzyme immunoassay—using an antibody that binds to all major prostaglandin compounds, results are expressed in percent of inhibition, calculated according to kit instructions.

### 4.7. Cytotoxicity Evaluation

Cytotoxicity of the extracts was evaluated by measuring the viability of Human Cardiac Microvascular Endothelial Cells (HCMEC) obtained from the American Type Culture Collection (ATCC). Cells were seeded in a 96-well plate with a final concentration of 10 × 10^4^ cells mL^−1^ with Dulbecco’s Modified Eagle Medium (DMEM) (Sigma-Aldrich (St. Louis, MO, USA) for 24 h.

The cellular viability was assessed by the mitochondrial-dependent reduction of 3-(4,5-dimethylthiazole-2-yl)-2,5-diphenyltetrazolium bromide (MTT) (Sigma-Aldrich (St. Louis, MO, USA) to formazan, quantified by optical density measurement at 510 nm, as described by Lopes et al. [74]. Several concentrations of the extracts were tested: 50, 100, 200, and 300 μg·mL^−1^—using DMSO 1% as negative control and DMSO 20% as the positive control. The assay was independently repeated four times, with duplicate extracts. Cytotoxicity was expressed as a percentage of cell viability, considering the values of the negative control as 100% viability.

## Figures and Tables

**Table 1:** Fatty acid concentration (μg_Fatty Acid_·mg_Extract_^−1^) ± standard deviation and content (m_Fatty Acid/_m_Total Fatty Acid_, *%*) in each *Gloeothece* sp. extracts, E—ethanol extract, A—acetone extract; HI (3:2)—Hexane:Isopropanol (3:2, *v*/*v*) extract, and EL—ethyl lactate, in terms of monounsaturated fatty acids (MUFA) and polyunsaturated fatty acids (PUFA).

Fatty Acids	Fatty Acids Concentration and Content (μg_FA_·mg_E_^−1^, %(m_FA_/m_TFA_)			
	E	A	HI (3:2)	EL
C14:1	0.520 ± 0.002	1.495 ± 0.013	0.937 ± 0.001	0.607 ± 0.020
	0.3	1.3	2.3	3.3
C16:1	1.046 ± 0.053	2.426 ± 0.158	0.994 ± 0.023	0.869 ± 0.092
	0.7	1.7	2.7	3.7
C17:1	6.849 ± 0.012	19.154 ± 2.152	2.517 ± 0.099	1.017 ± 0.187
	4.3	5.3	6.3	7.3
C18:1 n9 c+t	22.812 ± 1.118	53.796 ± 2.918	12.910 ± 2.598 ^a^	12.767 ± 1.980 ^a^
	14.4	15.4	16.4	17.4
C22:1 n9	0.184 ± 0.010 ^a^	0.849 ± 0.043	0.202 ± 0.057 ^a^	0.317 ± 0.016
	0.3	0.2	2.3	3.3
Σ MUFA	31.412	76.870	17.559	15.577
	20.0	22.4	8.5	15.6
C18:2 n6 t	24.242 ± 0.597	59.711 ± 3.278	11.683 ± 1.432	6.240 ± 1.510
	15.3	16.3	17.3	26.4
C18:2 n6 c	0.406 ± 0.025	0.984 ± 0.012	0.308 ± 0.083 ^a^	0.337 ± 0.008 ^a^
	0.3	1.3	2.3	3.3
C18:3n6	1.934 ± 0.030	1.467 ± 0.039 ^a^	1.250 ± 0.152 ^b^	1.267 ± 0.196 ^a,b^
	1.2	2.2	3.2	4.2
C18:3 n3	37.233 ± 0.685	96.765 ± 5.713	13.216 ± 0.225	4.575 ± 1.437
	23.4	24.4	25.4	18.3
C20:2	0.498 ± 0.009	0.724 ± 0.205 ^a^	0.943 ± 0.701 ^a^	0.289 ± 0.014
	0.25	1.3	2.3	3.3
C20:5 n3	0.344 ± 0.023 ^a^	-	0.283 ± 0.105 ^a^	0.462 ± 0.071
	0.2	-	2.2	3.2
Σ PUFA	64.160	159.651	27.682	13.170
	40.7	46.0	11.5	13.2

^a,b^ Same lowercase letters for the same fatty acid mean no significant difference between extracts (*p* < 0.05).

**Table 2:** Comparison of antioxidant capacity of *Gloeothece* sp. extracts (average ± standard deviation), against the radicals ABTS^+•^, DPPH^•^, ^•^NO^−^ and O_2_^•−^, expressed in terms of IC_50_ (mg_Extract_·mL^−1^), and values of IC50 values (average ± standard deviation) of extracts on cell viability, according to sulforhodamine B (SRB) assay for gastric cancer cell lines, AGS.

Solvents	Antioxidant Capacity IC_50_ (mg_E_·mL^−1^)				SRB IC_50_ (µg_E_·mL^−1^)
	ABTS^•+^	DPPH^•^	O_2_^•−^	^•^NO^−^	
Ethanol	0.259 ± 0.074 ^a,b^	1.538 ± 0.012	nd	0.637 ± 0.024	241.0 ± 22.5 ^a^
Acetone	0.217 ± 0.009 ^a^	0.978 ± 0.032	nd	0.284 ± 0.090	114.4 ± 6.4
HI 3:2 (*v*/*v*)	0.283 ± 0.034 ^b^	nd	nd	1.258 ± 0.353	23.2 ± 1.9
Ethyl lactate	5.809 ± 0.203	4.016 ± 1.256	nd	nd	209.3 ± 11.0 ^a^

^a,b^ Means within the same column, without a common superscript, are significantly different (*p* < 0.05). HI—Hexane: isopropanol (3:2) *v*/*v*; nd—not determined.

**Table 3:** Anti-inflammatory potential of *Gloeothece* sp. lipidic extracts, upon the protection of HRBC membranes (average ± standard deviation) from heat, expressed in percentage of stabilization and IC50 (average ± standard deviation) values of extracts obtained at of COX-2 enzymatic activity inhibition.

Solvents	HRBC Stabilization (%)	COX-2 Enzymatic Activity Inhibition IC_50_ (µg_E_·mL^−1^)
Acetone	-	116.8 ± 7.7
Ethanol	-	198.3 ± 15.2
HI 3:2 (*v*/*v*)	61.6 ± 9.2	130.2 ± 7.4
Ethyl lactate	14.8 ± 4.3	-
